# High-Temperature Failure Evolution Analysis of K-Type Film Thermocouples

**DOI:** 10.3390/mi14112070

**Published:** 2023-11-07

**Authors:** Yong Ruan, Jiaheng Li, Qian Xiao, Yu Wu, Meng Shi

**Affiliations:** 1 Department of Precision Instruments, Tsinghua University, Beijing 100084, China; 2 Department of Electronic Information, Beijing Information Science and Technology University, Beijing 100192, China; 18611174495@163.com (J.L.); xiaoqian_mys@163.com (Q.X.); 3 Qi Yuan Laboratory, Beijing 100094, China; 18811346921@163.com; 4 MEMS Institute of Zibo National High-Tech Industrial Development Zone, Zibo 255000, China; shimqwe@163.com

**Keywords:** NiCr/NiSi TFTCs, transient temperature measurement, failure analysis

## Abstract

Ni90%Cr10% and Ni97%Si3% thin-film thermocouples (TFTCs) were fabricated on a silicon substrate using magnetron sputtering technology. Static calibration yielded a Seebeck coefficient of 23.00 μV/°C. During staged temperature elevation of the TFTCs while continuously monitoring their thermoelectric output, a rapid decline in thermoelectric potential was observed upon the hot junction reaching 600 °C; the device had failed. Through three cycles of repetitive static calibration tests ranging from room temperature to 500 °C, it was observed that the thermoelectric performance of the TFTCs deteriorated as the testing progressed. Utilizing the same methodology, Ni-Cr and Ni-Si thin films corresponding to the positive and negative electrodes of the TFTCs were prepared. Their resistivity after undergoing various temperature annealing treatments was measured. Additionally, their surfaces were characterized using Scanning Electron Microscopy (SEM) and X-ray Photoelectron Spectroscopy (XPS). The causes behind the decline in thermoelectric performance at elevated temperatures were analyzed from both chemical composition and microstructural perspectives.

## 1. Introduction

In modern industrial production, numerous scenarios require precise measurement of localized temperatures. For instance, the detection of tool temperatures during cutting processes in machining operations enables real-time monitoring of tool conditions, prolonging tool lifespan and reducing production costs [1,2,3,4]. In the realm of aviation engine systems, as thrust-to-weight ratios increase, the introduction of cooling air to lower temperatures becomes necessary. However, this cooling process can adversely affect engine efficiency. Consequently, temperature sensors are indispensable for obtaining accurate operational temperatures. Not only do these sensors serve as a basis for enhancing engine efficiency, but they also facilitate the real-time monitoring of engine operational status [5,6,7,8,9].

Compared with traditional temperature measurement methods, such as wire-style thermocouples (WSTCs) and infrared radiation thermometers, thin-film thermocouples (TFTCs) can be deposited directly onto the surface of the object being measured without disrupting the device’s structure, causing minimal impact on the temperature field and gas flow over the device surface, while also offering faster response times [1,2,3,4,5,6,7,8,9].

TFTCs can be divided into two main categories: those composed of two different metallic materials, such as W-5Re/W-26Re and Pt-10Rh/Pt, and those composed of two semiconductor materials, like ITO/In_2_O_3_ and La_x_SR_1-x_CrO3 (LSCO)/ITO. Due to the high melting point and thermal stability of semiconductor materials, this kind of TFTC typically operates at higher working temperatures, and its output thermoelectric potential at the same temperature is higher than that of metallic material TFTCs. However, metallic material TFTCs offer higher temperature measurement accuracy and faster response times [5,10]. 

However, when traditional wire-strain thermocouples made of metal wires are transformed into thin-film thermocouples in the form of thin metal films using micro-electro-mechanical Systems (MEMS) technology, the devices experience varying degrees of stability degradation due to the significant reduction in size and thickness. They are more susceptible to performance degradation or even failure in high-temperature environments, and their operating temperature range is lower [11,12,13]. For instance, as Ruan’s article pointed out, TFTCs composed of W-5Re and W-26Re alloys exhibit good measurement accuracy and repeatability within the temperature range of −40 °C to 500 °C, along with very rapid response times. However, even though tungsten–rhenium alloys have high melting points and are coated with an SiC protective layer on TFTCs, they still experience failure when the environmental temperature reaches 620 °C, leading to a loss of thermoelectric output. This failure in TFTCs is attributed to horizontal oxidation diffusion, as observed in the failed TFTCs in [10]. In Zhao’s study, the microstructural evolution of S-type TFTCs composed of Pt-10Rh and R-type TFTCs composed of Pt-13Rh was investigated at high temperatures. After one hour of annealing at temperatures ranging from room temperature to 1000 °C, they observed rhodium segregation and oxidation on the thin-film surface, as well as a decrease in the Seebeck coefficient for both TFTC types, indicating a degradation in thermoelectric performance [14]. Kreider deposited TFTCs consisting of platinum (Pt) and palladium (Pd) onto silicon wafers with a silicon dioxide isolation layer by magnetron sputtering. These TFTCs exhibited high-temperature accuracy and had a working lifetime of 10–20 h at 850 °C. However, the research revealed that the stability of the palladium film was inferior to that of platinum. Palladium was more susceptible to oxidation in high-temperature environments, and at temperatures reaching 880 °C, it would coalesce and form pores, ultimately leading to the failure of the TFTCs [15]. K-type thermocouples, comprising Ni-Cr and Ni-Si alloys, are well-known for their reliable thermoelectric performance, excellent linearity, strong resistance to oxidation, and cost-effectiveness. They are widely used as cost-effective metallic thermocouples. When configured as wire-type thermocouples, they can operate continuously within the temperature range of 900 °C to 1000 °C [1,11]. However, corresponding Ni-10Cr and Ni-Si TFTCs generally exhibit limited functionality at higher temperatures. For instance, as shown in Zhang’s research, K-type TFTCs prepared by them exhibited erratic temperature–electromotive force curves at 400 °C, rendering them unsuitable for temperature measurement. At 450 °C, these TFTCs experienced a complete loss of thermoelectric potential output, resulting in failure [13].

In this study, K-type thin-film thermocouples were fabricated on silicon substrates using magnetron sputtering technology. During the testing of the TFTCs, with increasing temperature, performance degradation was observed, eventually leading to failure. To conduct a more comprehensive analysis of this process, Ni-Cr and Ni-Si thin films corresponding to the two junctions of the TFTCs were prepared using the same fabrication method. These thin films were subjected to thermal treatments of various temperatures in an ambient air atmosphere, and their resistivity was measured. The surfaces of the thin films were characterized using Scanning Electron Microscopy (SEM) and X-ray Photoelectron Spectroscopy (XPS). The TFTCs underwent staged temperature elevation and three rounds of repetitive static calibration testing. The causes underlying the thermal performance degradation of the K-type TFTCs are discussed from the perspectives of chemical composition and microstructural analysis.

## 2. Materials and Methods

Both WSTCs and TFTCs are based on the Seebeck effect. Equation (1) represents the relationship between the output voltage and temperature, where *S_A_* and *S_B_* represent the Seebeck coefficients of the two materials, respectively. *T*_1_ represents the temperature at the cold end, *T*_2_ represents the temperature at the hot end, and *V* represents the output thermoelectric potential [16].

(1) $$V=\int_{T1}^{T2}\left(SA-SB\right)Tdt$$

The Seebeck coefficient (*S*) of metallic materials is associated with fundamental physical quantities, such as the Fermi energy level, effective mass, relaxation time, and scattering mechanisms. When there is no temperature gradient, the Seebeck coefficient (*S*) is described as shown in Equations (2) and (3).

(2) $$S=\mp \frac{1}{eT}\left[\frac{K1}{K2}-EF\right]$$

(3) $$Ki=\int_{0}^{\infty }\tau u_{x}^{2}g\left(E\right)E^{i-1}\frac{\partial f_{0}}{\partial E}dE,\left(i=1\sim 3\right)$$

where *f*_0_ represents the distribution function of charge carriers at equilibrium, *h* is the characteristic parameter during relaxation processes, *E_F_* denotes the Fermi energy level, *τ* stands for the dielectric constant, and e represents the charge of a single electron. *g*(*E*) represents the carrier density of states near the conduction band bottom, as depicted in Equation (4):

(4) $$g\left(E\right)=\frac{4\pi {\left(2m^{*}\right)}^{\frac{3}{2}}}{h^{3}}E^{\frac{1}{2}}$$

where m* represents the effective mass of carriers, *h* stands for the Planck constant, and *E* denotes the energy of carriers. Whether composed of two metallic materials or two semiconductor materials, thermocouples operate based on the Seebeck effect, although their mechanisms of action are slightly different. Semiconductor material thermocouples, such as ITO-In_2_O_3_, form p-n junctions at their intersections, and the Seebeck coefficients of the two materials need to be considered separately. However, in the context discussed here, the metal type does not form a p-n type junction. Instead, metallic thermocouples operate in pairs for both materials, leading to distinct free electron diffusion rates and a single Seebeck coefficient for each component. For instance, W-5Re and W-26Re are utilized separately, and they are typically not individually characterized [9].

A 6-inch diameter silicon wafer with a thickness of 0.4 mm was utilized as the substrate. To avert alterations in the conductivity performance of silicon wafers at elevated temperatures and the generation of silicides via interactions with metallic films, it is imperative to employ an insulating material to establish electrical and reactive isolation between the TFTCs and the silicon substrate [15,17]. In this context, we opted for SiO_2_ as our chosen insulating material.

**Figure 1:**
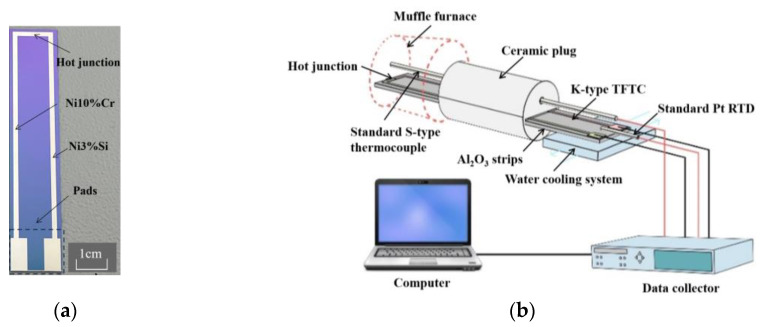
(**a**) Photograph of the K-type (Ni10%Cr–Ni3%Si) TFTCs; (**b**) calibration set-up of the TFTCs.

We initiated the fabrication process by growing a 500 nm SiO_2_ layer on silicon wafers using Plasma-Enhanced Chemical Vapor Deposition (PECVD), resulting in a distinct blue coloration [15]. Subsequently, the substrates underwent ultrasonic cleaning in pure ethanol and acetone baths, followed by nitrogen purging for desiccation, aimed at the removal of surface contaminants and moisture. The anode (left leg) of the TFTCs was initially prepared by depositing a 400 nm thick Ni-Cr film using DC magnetron sputtering. A positive photoresist (AZ 5214) was then spin-coated onto the surface. Employing ultraviolet photolithography and development processes, patterning was achieved, selectively retaining the photoresist only over the TFTCs’ anode region. The exposed Ni-Cr film beyond the photoresist-protected area was subsequently etched using chromic acid solution. After cleaning, the residual photoresist was removed using acetone. This procedure was iterated to fabricate the cathode (right leg) of the TFTCs. High-purity (99.997 wt.%) 100 mm diameter Ni-10%Cr and Ni-3%Si discs were employed as the sputtering targets. The specific sputtering parameters are detailed in Table 1. The overall length of the thin-film thermocouple was 8 cm, with each leg having a width of 1.5 mm. The overlapping node width between the Ni-Cr and Ni-Si lines was also 1.5 mm. The prepared TFTCs are depicted in Figure 1a. Using identical sputtering parameters and target materials, 400 nm thick Ni-Cr and Ni-Si films were deposited onto two silicon substrates of the same nature (also featuring a 500 nm SiO_2_ layer). These films were subsequently divided into 1 cm × 1 cm square samples.

All Ni-Cr and Ni-Si thin films underwent a thermal treatment in an ambient air environment at various temperatures for a duration of 30 min. The heating rate employed was 5 °C per minute, followed by natural cooling. The resistance of the thin film samples was measured using a four-point probe resistance measurement system. To characterize the surface chemical properties and microstructure of the thin films, XPS and SEM were employed. XPS provided insight into the chemical composition of the film surfaces, while SEM allowed for the observation of the microstructural features. XPS analysis yielded valuable information regarding the elemental composition and chemical states present on the thin film surfaces. SEM imaging facilitated the investigation of the microscale morphology and structural characteristics of the films.

The static calibration and testing of the TFTCs are illustrated in Figure 1b. The TFTCs were affixed onto Al_2_O_3_ ceramic strips using a high-temperature adhesive. By utilizing high-temperature silver paste, the copper wires were securely affixed to the pad, enabling the collection of thermoelectric output from the TFTCs. The hot junctions of the TFTCs were positioned within a muffle furnace, while the pads were located externally. A customized ceramic plug with an aperture and a cooling water circulation system were used to amplify the temperature difference between the hot junctions and the pad. The temperature of the TFTCs’ hot junctions was measured using a standard type-S thermocouple (with a nominal accuracy of ±0.5 °C), and the temperature at the pads (i.e., the cold junction temperature) was measured using a standard platinum resistance temperature detector (RTD) (with a nominal accuracy of ±0.1 °C). All data were collected and recorded on a computer using a data collector. The internal software in the data collector that converted resistance or voltage measurements into equivalent temperature had a nominal mathematical conversion accuracy of ±0.05 °C for thermocouples and ±0.02 °C for platinum RTD sensors. The TFTCs underwent calibration through several thermal cycles, and each calibration temperature was maintained for a minimum of 15 min to achieve thermal equilibrium.

## 3. Results

### 3.1. Characterization and Testing of the NiCr and NiSi Thin Films

**Figure 2:**
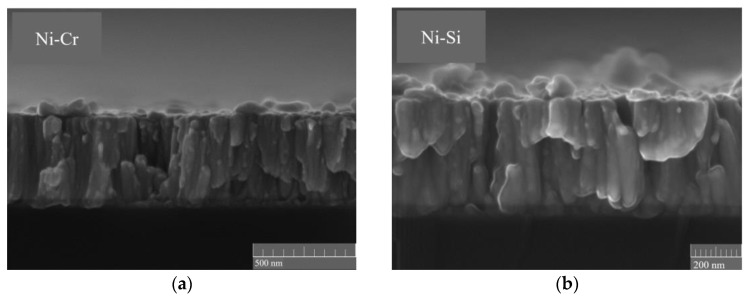
The cross-section SEM images of the (**a**) Ni-Cr and (**b**) Ni-Si thin films.

**Figure 3:**
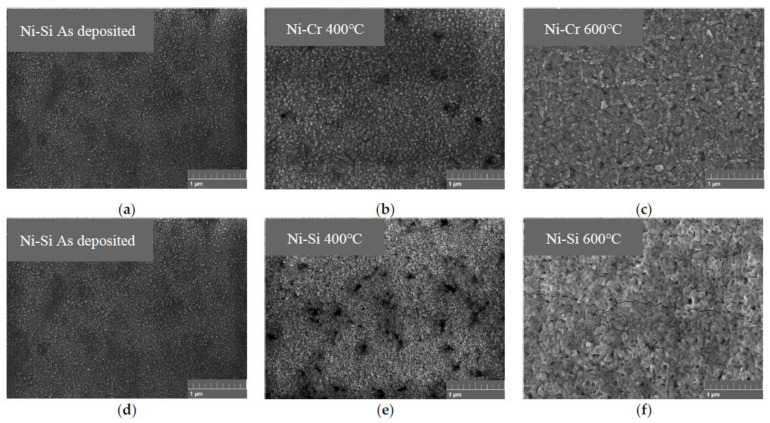
The surface SEM images of Ni-Cr and Ni-Si thin films annealed at different temperatures. (**a**,**d**) As deposited, (**b**,**e**) 400 °C, (**c**,**f**) 600 °C.

Figure 2 and Figure 3 illustrate the characterization of the Ni-Cr and Ni-Si thin films subjected to various temperature annealing processes using SEM. In our process, a scribe pen was employed to gently create a scribe mark on the backside of the silicon wafer (the side without deposited Ni-Cr and Ni-Si thin films). Subsequently, a controlled force was applied to induce the fracturing of the silicon wafer while endeavoring to preserve the cross-sectional structure of the Ni-Cr and Ni-Si thin films as much as possible. In Figure 2, it can be observed that both types of deposited thin film exhibit slight columnar structures at their cross-sections. As shown in Figure 3a,d, the surfaces of both types of thin film without annealing demonstrate a dense morphology without cracks or pore-like defects. The surface structure of the Ni-Cr thin film appears more refined, similar to the morphology characteristics observed in thin films prepared by W. Tillmann [18].

When the annealing temperature reached 400 °C, as depicted in Figure 3b,e, the surfaces of the two types of film became rougher with a stronger grainy texture, attributed to the result of annealing causing grain growth [11,14]. Some darker-colored oxidation regions appeared on the surfaces of both types of thin film, with fewer oxidation regions observed on the surface of the Ni-Cr thin film. For the Ni-Si thin film, due to the significantly smaller size of Si atoms/ions compared with Ni atoms/ions, it is an interstitial solid solution [19,20]. During the annealing process, impurities (Si atoms) tend to segregate at grain boundaries. These segregated Si atoms readily undergo chemical reactions with O_2_ in the air. It has been postulated by Q. Zhang that the oxidation process of the Ni-Si film initiates from these grain boundaries enriched with Si segregation, forming dispersed oxidation regions, as observed in Figure 3e, which gradually propagate throughout the entire thin film [13].

As the annealing temperature reached 600 °C, as illustrated in Figure 3c,f, for Ni-Si thin films, the oxidation zone diffused throughout the entire film, resulting in a surface consisting entirely of aggregated oxide islands and the appearance of cracks. In contrast, the Ni-Cr thin film remained crack-free. This discrepancy could be attributed to the incorporation of Cr elements, which likely enhanced the mechanical properties of the film. This improvement includes an increase in film hardness and enhanced adhesion to the substrate [18,21]. The oxidation process of Ni-Cr and Ni-Si thin films commences from localized oxidation regions and eventually spreads across the entire film surface. During this process, the Ni-Cr thin film exhibits lower oxidation levels and better integrity compared with the Ni-Si thin film, showing superior antioxidation capabilities and mechanical performance.

**Figure 4:**
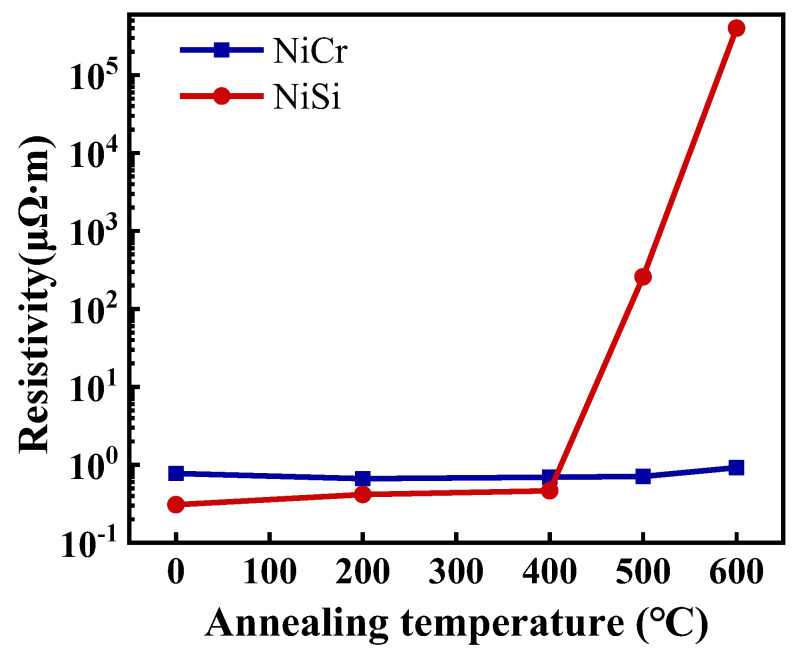
The resistivity of Ni-Cr and Ni-Si films annealed at different temperatures.

The electrical resistivity of a thin film is correlated with its Seebeck coefficient, and it can indirectly provide insights into issues such as thin film oxidation, mechanical stress, and crack formation [13,15]. In comparison to measurements of other related phenomena, the measurement of electrical resistivity offers a more expedient and simplified approach. In this study, we utilized a four-point probe electrical resistance measurement apparatus to assess the electrical resistivity of Ni-Cr and Ni-Si thin films that had undergone distinct temperature-based annealing treatments, as shown in Figure 4.

Prior to undergoing thermal treatment, the electrical resistivity of the Ni-Si thin films was marginally lower than that of the Ni-Cr thin films, which aligns with the respective conductive properties of these materials. On the one hand, in comparison to nickel, chromium is a material with relatively inferior electrical conductivity [18]. On the other hand, according to Matthiessen’s rule [22], the specific resistivity of a thin film (ρF) is the sum of scattering contributions from the ideal bulk lattice (ρB), the film’s surface (ρS), and imperfections (ρI). Compared with pure Ni thin films, Ni-Cr thin films display reduced crystallite sizes, potentially leading to heightened scattering at defects and subsequently contributing to an elevated electrical resistivity in the Ni-Cr thin film [18,22,23].

When the Ni-Si thin films were subjected to annealing temperatures of 400 °C and below, their resistivity remained at 10^−1^ μΩ∙m. However, when the annealing temperature reached 500 °C, the resistivity abruptly increased to 258 μΩ∙m. At 600 °C, the resistivity exceeded the measurement instrument’s upper limit, reaching 10^5^ μΩ∙m. Cracks observed on the surface of the Ni-Si thin films in Figure 3e may be one of the factors contributing to this sharp increase in resistivity. In contrast, the resistivity of the Ni-Cr thin films consistently remained at 10^−1^ μΩ∙m. It is evident that within the 600 °C range, the Ni-Cr thin films exhibited greater stability. This stability relied on the formation of a thin oxide layer with a thickness of approximately 2 nm [18,24].

**Figure 5:**
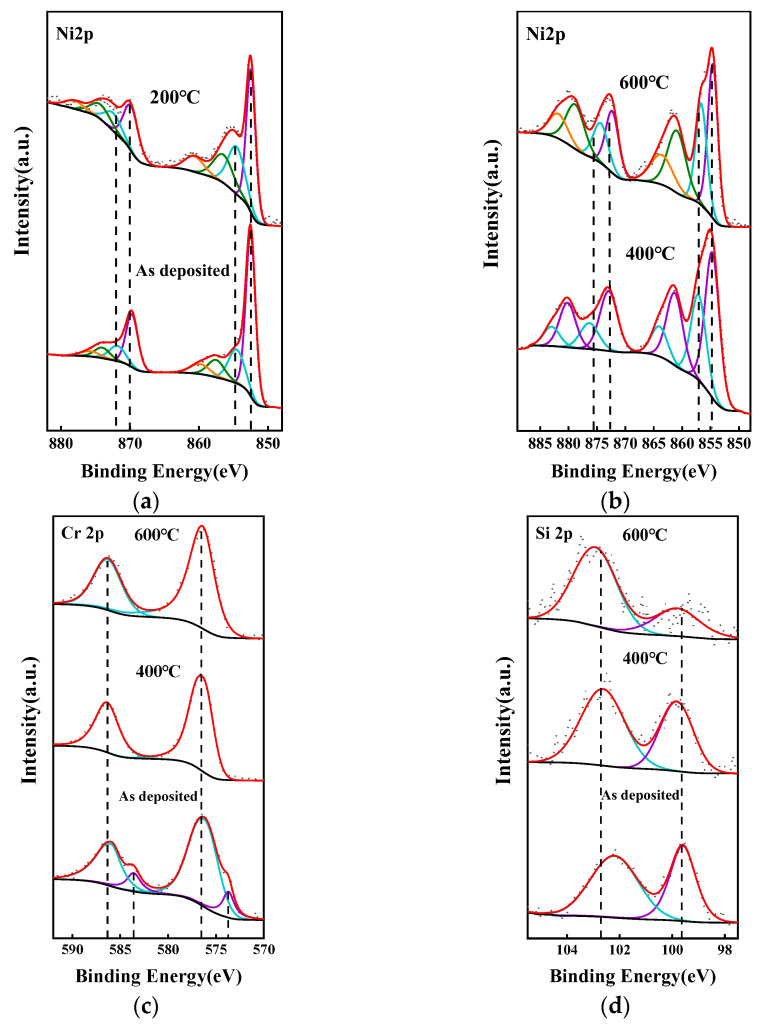
The high-resolution XPS spectra of (**a**,**b**) Ni 2p, (**c**) Cr 2p, and (**d**) Si 2p of Ni-Cr and Ni-Si thin films annealed at different temperatures.

Figure 5 presents the XPS spectra of the Ni-Cr/Ni-Si thin film samples after undergoing annealing processes at various temperatures, with calibration performed using the binding energy of C 1s at 284.8 eV.

Figure 5a,b displays the XPS spectrum of the nickel element. For nickel, the peak shapes in the detection results for both alloy films are similar. The data presented here are from the Ni-Cr thin film. The Ni 2p region displays four readily distinguishable characteristics: the prominent Ni 2p3/2 peak, along with its satellite, located at approximately 854 and 862 eV, in addition to the primary Ni 2p1/2 peak and its accompanying satellite, positioned around 872 and 879 eV. The underlying electronic structure exhibits increased complexity, reflecting the strongly correlated electronic nature of the valence band. Typically, each spin-orbit multiplet is decomposed into multiple supplementary components, as depicted for the Ni 2p3/2 and 2p1/2 regions, where a minimum of four peaks is necessary to accurately replicate the observed peak shape [25]. As the annealing temperature increases, a split peak emerges near 855 eV in the 2p 3/2 region, with its intensity gradually escalating. The peaks’ positions shift to higher binding energies following annealing at 400 °C and 600 °C, indicating a more extensive surface oxidation of the samples [13,25].

Figure 5c,d depict the XPS spectra of the chromium and silicon elements, respectively, in the Ni-Cr and Ni-Si thin films after undergoing annealing processes at different temperatures. Regarding the chromium elements, as the annealing progresses, the split peaks located around 574 eV and 584 eV in the Cr 2p3/2 and 2p1/2 regions, respectively, disappear, indicating the oxidation of chromium elements on the sample surface [26]. For silicon elements, with the increase in annealing temperature, the intensity of the peak near 99.6 eV gradually decreases, signifying an increasing degree of silicon element oxidation [13].

The EDS analysis of the Ni-Cr thin film is presented in Table 2. It is evident that with an increase in annealing temperature, the proportion of oxygen atoms within the film significantly rises, indicating a progressive deepening of film oxidation. Notably, even in the absence of annealing, the unprocessed thin film samples exhibit an oxygen atom ratio of approximately 9.83%. This suggests inherent oxidation in the samples, likely stemming from the natural oxidation in the atmospheric environment at room temperature and the repeated utilization of target materials, thereby causing a self-oxidation phenomena [11,24].

### 3.2. Thermoelectric Properties of Ni-Cr and Ni-Si Thin-Film Thermocouples

**Figure 6:**
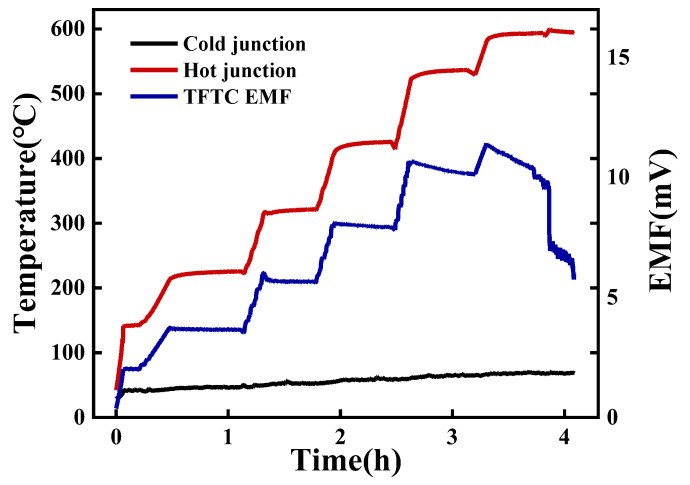
Staged temperature elevation results of the TFTCs.

The TFTCs were subjected to a staged temperature elevation and their output electromotive force (EMF) was continuously measured. As depicted in Figure 6, it is evident that at hot-end temperatures below 420 °C, a robust correlation exists between the output EMF and variations in temperature. Upon the attainment of a hot-end temperature of 530 °C during the insulation phase, a marginal decrement in EMF is discernible, indicating an appreciable diminishment in thermoelectric performance. Upon the temperature reaching 600 °C, the EMF experiences an abrupt and precipitous reduction, rendering the continuation of measurements unattainable, thereby signifying the failure of the TFTCs. This is highly analogous to the trend of resistivity variation in Ni-Si thin films with heat treatment, as depicted in Figure 4. The failure of the TFTCs is primarily attributed to the oxidation and cracking of the comparatively more fragile negative electrode Ni-Si thin films within the temperature range of 500 °C to 600 °C. This limitation hampers the operational capabilities of K-type TFTCs at elevated temperatures.

The TFTCs underwent repeated static calibrations, where the hot junction was elevated from room temperature to 500 °C, followed by natural cooling. This process encompassed three heating–cooling cycles. Each calibration temperature was maintained for a minimum of 15 min to ensure thermal equilibrium before data collection commenced. Equation (5) is used to describe the thermoelectric behavior of TFTCs:

(5) $$E\left(T^{*}\right)=A\times {\left(T^{*}\right)}^{2}+B\times \left(T^{*}\right)+C$$

where T* represents the temperature difference between the cold junction and hot junction, measured in degrees Celsius (°C). *E* represents the output thermoelectric potential, measured in millivolts (mV). To account for practical experimental conditions where the output thermoelectric potential (*E*) is zero when the temperature difference (T*) is zero, the parameter *C* is set to zero as a boundary condition in the equation.

**Figure 7:**
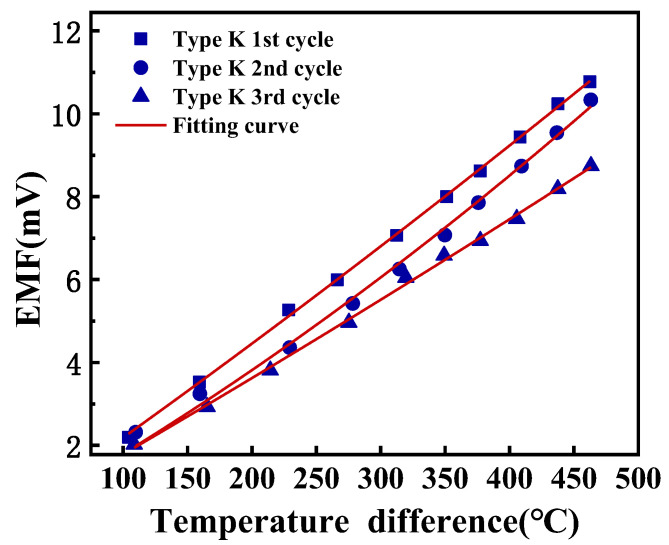
The repetitive static calibration test result of K-type TFTCs.

The quadratic fitting outcomes and the averaged Seebeck coefficient of each testing round are presented in Table 3. All the fitting coefficients had an R^2^ > 0.995. As discernible from Figure 7, at identical temperature points, the output thermoelectric potential exhibited a diminishing trend, with the fitted EMF curve progressively declining. This indicates a degradation in the thermoelectric performance of the TFTCs and a reduction in sensitivity. Furthermore, as the testing progressed, an increasing number of points began to deviate from the fitted curve. This indicates a deterioration in the linearity and operational stability of the TFTCs.

The Seebeck coefficient of the TFTCs is notably lower than that of the K-type wire-style thermocouple (WSTC), which is 42.3 µV/°C [11]. This difference may have arisen from two distinct aspects. First, for pure metallic thin films, the electronic thermopower (also called Seebeck coefficient) can be expressed as follows (Equations (6) and (7)):

(6) $$S_{F}=S_{B}\left[\left(\frac{5}{8}\right)\left(\frac{l}{t}\right)\left(1-p\right)\frac{U}{1+U}\right]\left(t\gg l\right)$$

(7) $$U={\left(\frac{\partial \ln l}{\partial \ln E}\right)}_{E=\zeta }$$

where *S*_F_ is the electronic thermopower of the film, *S*_B_ is the electronic thermopower of the bulk material, *l* is the mean free path of carriers, *t* is the film thickness, *p* is the scattering coefficient, and *ξ* is the Fermi energy [27]. In comparison to bulk materials, thin-film materials exhibit smaller grain sizes, leading to an increase in grain boundaries. Defect segregation at grain boundaries may create additional potential barriers, inhibiting the movement of charge carriers towards grain boundaries [11]. This results in a reduction of the electron mean free path, typically within the range of only 10^0^ to 10^1^ nm [24,28], ultimately leading to a decrease in the Seebeck coefficient of the TFTCs [11,29]: this is called the grain size effect. In the context of this study, the film’s thickness is 400 nm, and quantum size effects significantly influence the behavior only when the thin film thickness is comparable to the average free path of its carriers [30,31].

On the other hand, the hot junction structure of the TFTCs prepared in this study involves a negative-pole Ni-Si thin film deposited onto a positive-pole Ni-Cr thin film. Both films have a thickness of 400 nm, and the overlapping section between them has a length of 1.5 mm. Electrons within the positive-pole Ni-Cr thin film can only move into the Ni-Si thin film through the overlapping region. Due to the constraints posed by the thin film’s thickness, the positive-pole film cannot supply a sufficient number of free electrons to transition effectively to the negative-pole film. This leads to a lower output thermoelectric potential (EMF) at the same temperature compared with the WSTC, resulting in a lower Seebeck coefficient in the static calibration results [28].

## 4. Conclusions

In this study, we used magnetron sputtering to create K-type TFTCs along with corresponding Ni90%Cr10% and Ni97%Si3% thin-film samples for the positive and negative poles, employing the same procedure. As we raised the thermal treatment temperature for the thin-film samples, surface element oxidation was observed. The film surface structure transitioned from dense to scattered oxidized areas and eventually consolidated into aggregated oxide islands. Cracks emerged on the Ni-Si thin film’s surface, potentially causing its sharp resistance increase. Conversely, the Ni-Cr thin film’s resistance remained stable at 10^−1^ μΩ∙m. This difference underscores Ni-Si’s increased brittleness compared with Ni-Cr within the 600 °C temperature range. After undergoing static calibration with a maximum hot junction temperature of 500 °C, the TFTCs exhibited a Seebeck coefficient of 23.00 µV/°C, significantly lower than that of standard K-type metal wire thermocouples. With the repetition of static calibration, it was observed that the TFTCs experienced a decrease in thermoelectric potential at the same temperature points, indicating a degradation in thermoelectric performance. During staged temperature testing, it was found that the TFTCs’ thermoelectric performance started to decline as the hot-end temperature reached 530 °C. At 600 °C, failure occurred. This closely parallels the temperature-dependent variation trend of Ni-Si thin film resistance, indicating that a more fragile Ni-Si film constrains the working capacity of K-type TFTCs in high-temperature environments.

## Figures and Tables

**Table 1:** Sputtering parameters of NiCr/NiSi film.

Material	Target-Base Distance (mm)	Background Pressure (Torr)	Working Pressing (Torr)	Sputtering Power (W)	Ar_2_ Flow Rate (sccm)
NiCr	65	5 × 10^−6^	6.1 × 10^−3^	180	25
NiSi	65	5 × 10^−6^	6.1 × 10^−3^	180	25

**Table 2:** EDS elemental analysis of Ni-Cr films.

Samples		Atomic %	
	Ni	Cr	O
Ni-10%Cr annealed at 600 °C	60.15	7.36	32.48
Ni-10%Cr annealed at 400 °C	72.47	9.37	18.17
Ni-10%Cr as deposited	80.22	9.95	9.83

**Table 3:** Polynomial fitting of thermoelectric performance of K-type TFTCs.

	Coefficients of Polynomial V(T) = A(∆T)^2^ + B∆T + C			
Thermocouple	A (mV/°C^2^)	B (mV/°C)	C (mV)	Average Seebeck Coefficient (µV/°C)
Type K 1st cycle	4.03 × 10^−6^	2.15 × 10^−2^	0	23.00
Type K 2nd cycle	1.09 × 10^−5^	1.69 × 10^−2^	0	21.04
Type K 3rd cycle	2.59 × 10^−6^	1.76 × 10^−2^	0	18.58

## Data Availability

Not applicable.
